# Editorial: The immunological effects of respiratory viruses during pregnancy and breastfeeding

**DOI:** 10.3389/fimmu.2025.1715204

**Published:** 2025-10-17

**Authors:** Stella Liong, K. H. Christopher Choy, Stavros Selemidis, Bahaa Abu-Raya, Domenico Umberto De Rose

**Affiliations:** ^1^ Centre for Respiratory Science and Health, School of Health and Biomedical Sciences, RMIT University, Bundoora, VIC, Australia; ^2^ Drug Discovery Biology, Monash Institute of Pharmaceutical Sciences, Monash University, Parkville, VIC, Australia; ^3^ Canadian Center for Vaccinology, Dalhousie University, Izaak Walton Killam (IWK) Health Centre and the Nova Scotia Health Authority, Halifax, NS, Canada; ^4^ Department of Pediatrics, Dalhousie University, Halifax, NS, Canada; ^5^ Department of Microbiology and Immunology, Dalhousie University, Halifax, NS, Canada; ^6^ Neonatal Intensive Care Unit, “Bambino Gesù” Children’s Hospital IRCCS, Rome, Italy

**Keywords:** pregnancy, respiratory viruses, early life development, cardiorespiratory system, breastfeeding, COVID-19

Pregnancy and the early postnatal period are marked by unique immunological adaptations that heighten susceptibility to infection, particularly from respiratory viruses. Dysregulation of maternal and fetal immunity during early gestation or soon after birth can have long-term health consequences. The recent COVID-19 pandemic has underscored the importance of understanding how respiratory viral infections perturb the finely tuned and balanced maternal-fetal immune environment during these critical developmental periods (1).

This Research Topic explores the immunological effects of respiratory viruses during pregnancy and breastfeeding, offering insights into maternal-infant crosstalk, infant immune development, clinical management and neurodevelopmental risks. Indeed, infections with respiratory viruses, including SARS-CoV-2, present distinctive challenges, as their effects extend beyond maternal morbidity to shape transplacental immune transfer, breastmilk composition, and long-term offspring health outcomes.

Despite initiation exclusion of pregnant women from clinical trials, multiple studies have now shown that COVID-19 vaccines are both safe and effective in pregnancy, facilitating transfer of maternal neutralizing antibodies via the placenta and breastmilk (2). Human breastmilk contains bioactive factors and immune cells that shape neonatal immunity, which can be further enhanced by maternal vaccination (3).

Canellas-de-Castro et al. complemented this perspective by profiling chemokines, cytokines, and growth factors in 141 paired maternal and cord blood samples across acute and convalescent phases of SARS-CoV-2 infection, along with 8 health controls. These samples were obtained between July 2020 to December 2021 in Brazil, during the circulation of the B.1.1.28 and B.1.1.33 SARS-COV-2 variants. They highlighted how increased levels of chemokines and cytokines (CCL11, IFN-γ, IL-1Ra, and G-CSF) in fetal cord blood were associated with maternal infection. This demonstrated the connection between the maternal and fetal immune microenvironment, showing how maternal infection affects newborn immunity in ways that go beyond simple antibody transfer alone.

In addition, Masry et al. investigated serologic profiles in newborns and mothers with previous COVID-19 infection or vaccination in Doha (Qatar) between 2021 to 2022. Maternal vaccination, particularly with three doses, led to higher neutralizing and spike receptor-binding domain antibody titers (NTAb*3.31 and S-RBD*1.15) in fetal cord blood compared to the infected but non-vaccinated group, highlighting the efficacy and importance of maternal vaccination in promoting the fetal immunity. This strengthens the rationale for recommending COVID-19 vaccine boosters during pregnancy.

Conversely, with later emerging variants of SARS-CoV-2, Govindaraj et al. showed that even the latest mRNA vaccinations had limited effect against Omicron (BA1, BA2, and BA4/5) variants, as evidenced by lower neutralizing antibodies levels despite robust overall antibody titers. This underscores the challenge posed by evolving viral variants and the need for updated vaccines to ensure the continuous protection for mothers and infants.

From a population-level perspective, the burden of respiratory infections during pregnancy was reported by Álvarez-del Río et al. in a nationwide Spanish analysis, which included over 779,000 childbirth cases. The authors found that COVID-19 positive mothers had increased risks of intensive care unit admission, ventilation/intubation, and in-hospital mortality. Women with COVID-19 had a significantly greater risk of postpartum hemorrhage (OR = 1.14), embolism (OR = 7.98), acute respiratory distress syndrome (OR = 35.5), temporary tracheostomy (OR = 4.89), ventilation/intubation (OR = 6.85), and single stillbirth (OR = 1.32) (p < 0.05). These pregnancy complications were associated with higher hospitalization costs and mortality, further demonstrating the importance of preventive strategies to mitigate their impact on healthcare systems.

From a public health perspective, breastfeeding should be strongly supported, where possible, because it provides an immunity crosstalk between the mothers and infants, with maternal antibodies offering passive immunity to neonates against pathogens, and by promoting the normal development of the infant mucosal immune system (4).

Zheng et al. demonstrated that respiratory infections in nursing infants - primarily bronchiolitis (84%) and pneumonia (5%) - stimulate an increase in cytokines and chemokines in human breastmilk, facilitating the recruitment and activation of lymphocytes. Although the study did not specify the viral etiology, the diagnoses were based on clinical symptoms such as cough, rhinorrhoea, and fever, and confirmed through chest radiography showing hyperinflation, atelectasis, or infiltration. These findings highlight the continuous support provided by maternal immunity through breastfeeding, nurturing the infants’ immunological responses to respiratory pathogens.

Finally, the potential long-term consequences of maternal respiratory infections on offspring neurodevelopment were addressed by Manti et al. in a review including the impact of prenatal influenza, SARS-CoV-2, RSV and other prenatal respiratory infections (SARS-CoV-1, Middle East respiratory syndrome, and rubella). Evidence suggests that viruses such as influenza and SARS-CoV-2 could perturb fetal brain development through vertical transmission or maternal immune activation (MIA). Both clinical and preclinical studies have demonstrated associations between MIA and neuropsychiatric disorders, including schizophrenia and autism spectrum disorder (5–7). The authors further emphasize the need for research into the mechanisms of neuroinflammation, its impact on developmental trajectories, and potential interventions.

In summary, this Research Topic offers insights on how respiratory viral infections intersect between maternal and neonatal immunity, through various common themes. First, maternal-infant immunity is extremely dynamic and multifaceted, involving not just antibody transfer but also specific mediator signaling and adaptive breastmilk responses. Second, while maternal vaccination remains a critical strategy, variant-driven immune escape, that is very relevant to SARS-CoV-2, presents continued hurdles. Third, mechanistic findings at the cellular and molecular levels are consistent with population-level data on obstetric hazards, highlighting the field’s translational value. Finally, the neurodevelopmental dimension emphasizes that the effects of maternal virus infection may persist well beyond the perinatal period. Other relevant Research Topics that might be of interest include: Updates on Immunity to Influenza A Virus in Humans and Animals; Immune Response to Respiratory Viruses and Respiratory Viral Infections in Susceptible Populations; How RSV Outsmarts the Host; and Viral Infection at the Maternal-Fetal Interface.

Looking ahead, research priorities include optimizing maternal vaccination strategies, potentially incorporating variant-specific booster regimens and evaluating their long-term neurodevelopmental benefits. Emerging, multi-omics approaches will help map maternal–fetal immune networks in greater detail, while longitudinal cohort studies will be essential to linking neonatal immune profiles with health outcomes throughout childhood. As new variants emerge and global health threats evolve, continued research is essential for developing new strategies and informing updated clinical guidelines and public health policies.
